# Intersectional inequalities in mental health by education, income, gender, and age before and during the COVID-19 pandemic in the Netherlands: a longitudinal study

**DOI:** 10.1186/s12939-024-02338-6

**Published:** 2024-11-25

**Authors:** Sanne E. Verra, Clare Evans, Joost Oude Groeniger, John de Wit, Maartje P. Poelman, Carlijn B. M. Kamphuis

**Affiliations:** 1 Department of Interdisciplinary Social Science, Utrecht University, Sjoerd Groenman building Padualaan 14, 3584CH, Utrecht, The Netherlands; 2 Department of Sociology, University of Oregon, Eugene, OR USA; 3 Department of Public Health, Erasmus MC University Medical Center Rotterdam, Rotterdam, The Netherlands; 4 Chair Group Consumption and Healthy Lifestyles, Wageningen University & Research, Wageningen, the Netherlands

**Keywords:** Intersectional framework, Health inequities, Social inequality, Mental health, Socioeconomic disadvantage in health, Gender inequality, Multilevel analysis, COVID-19, Longitudinal study

## Abstract

**Background:**

It remains unclear how COVID-19 has disproportionately affected the mental health of different vulnerable groups. This study explores how mental health inequalities changed between 2014 (pre-COVID-19) and 2021 (during COVID-19) in the Netherlands across intersectional social strata defined by interplays of educational attainment, income level, gender, and age.

**Methods:**

Using 2014 and 2021 self-reported cohort data on health and living conditions of the adult population of Eindhoven and surroundings (*N* = 1,157), a Multilevel Analysis of Individual Heterogeneity and Discriminatory Accuracy (MAIHDA) was applied to explore intersectional inequalities in mental health in 2014, 2021, and in mental health changes (2014–2021). We examined this using the Mental Health Inventory-5 across 53 intersectional social strata based on interplays of education, income, gender, and age in 2014.

**Results:**

There were substantial differences in mental health trajectories across social strata. Between-stratum mental health inequalities were patterned additively, indicating that inequality patterns along one axis (such as income) tended to be consistent across other axes of comparison. Additive trends revealed that women with a low income were at highest risk of poor mental health in 2014 and 2021, and people over 65 were at highest risk of mental health setbacks over time. Nonsignificant educational inequalities were found in 2014 and 2021. Income inequalities persisted, but slightly decreased in 2021 due to stronger mental health setbacks among those with high incomes. Women experienced persisting disadvantages that slightly flattened over time, and the mental health advantages of older age diminished over time.

**Conclusions:**

Inequalities in mental health add up for those who experience multiple axes of disadvantage, such as women and those with low incomes, but no disproportionate intersectional interaction effects were found. Effort is needed to ensure that mental health support is accessible for all, especially those with low incomes. Given the especially strong mental health declines among those over 65, responses to future crises need to include measures to protect the mental health of the elderly. Future research should investigate intersectional inequalities along other axes of disadvantage, such as ethnicity, employment and family status.

**Supplementary Information:**

The online version contains supplementary material available at 10.1186/s12939-024-02338-6.

## Background

There is a clear social gradient in mental health: people with vulnerabilities systematically experience poorer mental health compared to people who face fewer vulnerabilities [1, 2]. This may be the result of sustained inequalities in social conditions and available resources among vulnerable populations, such as quality housing, working conditions, and chronic stress [2–4].

When the COVID-19 pandemic hit, some anticipated that the pandemic would act as “the great equalizer” by affecting people across different societal positions similarly [5, 6], whereas others hypothesized that the pandemic would disproportionately affect vulnerable groups, exacerbating existing (mental) health inequalities and creating new ones [7–10]. National statistics revealed small decreases in overall population mental health in the Netherlands after the pandemic hit [11]. These setbacks in mental health likely had varying contributing explanations, such as the (fear of) negative health impacts due to COVID-19, losing loved ones, decreased social engagement, and facing financial and employment insecurities, all disproportionately affecting those least well off [12, 13].

Mental health may have suffered particularly among individuals facing added stress stemming from socioeconomic insecurity, such as job insecurity, financial instability, and precarious housing conditions [14]. When setbacks last, as they did during the COVID-19 pandemic, problems are likely to accumulate. According to the Vulnerability-Stress Model, individuals with pre-existing vulnerabilities, which may be more prevalent among those in a low socioeconomic position, are more susceptible to stress and subsequent mental health issues [15]. This would explain why people facing multiple, overlapping disadvantages, or collective traumas, may have been more likely to experience mental health declines during the pandemic [10]. Alternatively, based on the conservation of resources theory, it is also possible that people who had more to lose, such as those with high incomes or those with extensive social contacts, were at higher risk of mental health setbacks during the pandemic [15].

Many studies examined social indicators related to mental health changes in the context of COVID-19 pandemic, including education, income, gender, and age. For instance, van den Boom et al. [14] identified an educational gradient in mental health inequalities due to COVID-19 in the Netherlands. People with higher educational attainments likely experienced more control over their working situations, and had more cultural and social capital to counter some of the negative mental health effects resulting from the pandemic. However, other studies point towards a stronger influence of income rather than educational attainment in relation to mental health changes during COVID-19 [16–19]. People with lower incomes likely faced more (financial) barriers to ensure their basic needs, such as food and safe housing, in times of COVID-19 than people with higher incomes, since they were more likely to be in insecure and relatively exposed employment types, and to live in overcrowded homes [20].

Additionally, women were found to be at greater risk of overall deterioration in mental health during COVID-19 [17], due to gender inequalities in work, household and care responsibilities [21, 22]. Mental health declines during COVID-19 have also been suggested to vary by age. Adults below 40 years old experienced larger mental health decreases than population groups above 40, since they faced relatively larger setbacks in their social life compared to e.g., retired adults [17]. The negative influence on mental health among younger adults has been predicted to last going forward [14]. Meanwhile, the mental health of adults aged 65 or older seemed to remain relatively unchanged during COVID-19 [17]. Although this age group experienced relatively high health risks resulting from infection, they faced smaller setbacks in daily social and working lives, and have been hypothesized to have stronger coping skills and more resources (e.g., larger living spaces) due to their higher age [10, 14].

Socioeconomic factors, gender, and age are all relevant to consider when examining inequalities in mental health during COVID-19. However, while an interplay of overlapping disadvantages tied to one’s social conditions may have shaped mental health changes throughout the pandemic, social factors have mostly been studied in isolation.

Intersectionality is a critical theoretical framework proposed and developed by Black feminist scholars to draw attention to how individual (and collective) experiences are not shaped by separate, disconnected axes of disadvantage or advantage, but rather simultaneously by multiple interlocking systems of marginalization, oppression, and inequity [23, 24]. In relation to their mental health outcomes during COVID-19, each individual has a unique set of disadvantages (i.e., a low income level) and advantages (i.e., being aged between 40 and 65). Each unique set of (dis)advantages may have influenced individuals differently [9, 25, 26]. We should therefore consider how inequalities in health outcomes are patterned across intersectional positionalities and identities.

To our knowledge, one previous study by Moreno-Agostino et al. [27] used an intersectional lens to examine overlapping disadvantages and their joint influence on mental health during COVID-19. This study, however, had a cross-sectional design and was conducted in a different context. Moreno-Agostino et al. [27] examined the intersectional patterning of mental health issues during COVID-19 in the United Kingdom. Examining the interplay of age, gender, ethnicity, sexual orientation, and socioeconomic positions, they found some intersectional interaction effects among populations with specific combinations of privileges and marginalization. For instance, men in their 30s who identify as heterosexual and are of South Asian descent who were living in less deprived areas experienced a substantially lower life satisfaction during the pandemic than expected [27].

In this study, we explore intersectional inequalities in mental health before and during COVID-19, and changes over this time period, in the Netherlands based on educational attainment, income level, gender, and age. Understanding how mental health status has changed, and perhaps changed differently, for different population subgroups (or intersectional social strata) is essential to recognizing patterns of need and excess burden.

## Methods

### Data and study design

Cohort data from the GLOBE (Dutch acronym for “Health and Living Conditions of the Population of Eindhoven and surroundings”) study was used. GLOBE is a prospective cohort study initiated in 1991 in the Eindhoven area of the Netherlands that focuses on understanding socioeconomic inequalities in health. The area was strategically selected because the population was roughly representative of the Netherlands. The baseline study consisted of a postal survey sent out to a random sample based on municipal registries, stratified by age, degree of urbanization, and socioeconomic position, of non-institutionalized Dutch persons aged 14–75 years (response rate 70.1%, *N* = 18,973). Respondents were invited to postal surveys once every 6–7 years, and in several later waves of data collection, new samples have been added to compensate for attrition (including in 2014 and 2021).

Data from respondents who participated in the two most recent waves (2014 and 2021) were selected for this study. Both waves were collected between October and November, minimizing seasonal mental health differences between waves. In 2021, data was collected during the COVID-19 pandemic. After stringent infection control measures were put in place as of March 2020 and much of 2021, such as guidelines on social distancing and working from home as much as possible, measures were relaxed in October 2021 [28]. However, throughout November 2021, infection rates increased rapidly again, resulting in the reinstating of prior control measures and the introduction of new and more strict control measures, such as closures of non-essential stores and limits to the number of guests allowed at home [28]. Mental health data from the 2021 wave of data collection likely reflects the impact of the COVID-19 pandemic and mitigation measures.

A total of *N* = 1,354 respondents participated in both waves (2014 and 2021). For the main analyses, only participants with complete data (*N* = 1,157) were used. See Appendix 1 for an explanation of the sample selection and deletion of those with missing data. The use of personal data in the GLOBE study is in compliance with the Dutch Personal Data Protection Act and the Municipal Database Act; the study is registered with the Dutch Data Protection Authority (number 1248943). The analytic plan has been pre-registered at https://osf.io/a3kdx.

### Variables used in analyses

#### Mental health and change in mental health

Mental health was measured in 2014 and 2021 using the same five-item version of the ‘mental health inventory’ (MHI-5). The MHI-5 is validated in the Dutch general population [29–31], and consists of the following questions: (1) ‘Have you felt so down in the dumps that nothing could cheer you up?’, (2) ‘Have you felt downhearted and blue?’, (3) ‘Have you been a happy person?’, (4) ‘Have you been a very nervous person?’, and (5) ‘Have you felt calm and peaceful?’. Participants were asked how often they felt this way over the last four weeks, and were provided with six response options ranging from ‘all the time’ to ‘none of the time’. Data was used from those participants who answered at least three of the five items. After reverse coding the third and fifth questions, mental health scores for 2014 and for 2021 were calculated by taking the mean of the five items. These scores were then transformed to a 100-point scale by subtracting one point and multiplying by twenty to improve interpretation (a higher score indicating better mental health), in line with Noordzij et al. [32]. Cronbach’s alpha for the scale was 0.83 (2014) and 0.85 (2021), indicating high internal consistency. To measure the change in mental health score between 2014 and 2021, the 2014 mental health score was deducted from the 2021 mental health score, resulting in an absolute change in mental health score, potentially ranging from − 100 to 100. A negative score implies a setback in mental health between 2014 and 2021, whereas a positive score implies a mental health improvement between 2014 and 2021.

#### Intersectional social strata

Participants were assigned into their intersectional social strata based on 2014 data for education, income, gender, and age. Thus, 2021 inequality patterns between strata are based on the strata-cohorts that respondents belonged to in 2014.

##### Highest educational attainment

Participants reported their highest level of education completed. Three categories of educational attainment were included in the analysis. Categories were defined according to the International Standard Classification of Education (ISCED) as having a low educational attainment (and having either no diploma, or having completed primary education, or lower professional and intermediate general education (ISCED 0–2), intermediate educational attainment (intermediate professional and higher general educations; ISCED 3–4), and high educational attainment (higher professional education and university education; ISCED 5–7).

##### Household equivalent income

This was measured as the level of monthly household income divided by the square root of the number of people living from this income. This is a measure recommended by the OECD to adjust for the higher amount of income needed to sustain one person rather than a household with multiple persons [33]. Household equivalent income was divided into tertiles based on the following cut-off points: below or equal to 1500 euros per month, higher than 1500 and lower than or equal to 2200 euros per month, and higher than 2200 euros per month.

##### Gender

Two options were provided (“male” or “female”) and used in the analyses.

##### Age

Age was assessed in years and analyzed in three categories as follows (1) below 40 years old, (2) aged between 40 and 64, and (3) 65 or older in 2014. This means that data referring to 2021 or changes over time only captures participants in their thirties or older.

An intersectional social strata variable was created that represents every possible combination of educational attainment, income level, gender, and age (3 × 3 × 3 × 2 = 54 strata). One stratum was empty, as there were no young women with low educational attainment, combined with an intermediate income level in our data, resulting in 53 strata. Each intersectional social stratum was assigned a unique four digit ID code, the first digit represents educational attainment, the second digit represents income level, the third digit represents age group, and the final digit represents gender. For example, the digit 1111 represents those who shared a low educational attainment, a low income level, a young age (< 40 years old), and a female gender in 2014.

### Data analysis

An Intersectional Multilevel Analysis of Individual Heterogeneity and Discriminatory Accuracy (MAIHDA) was used, based on the approach of Evans et al. [34]. MAIHDA uses multilevel regression models. Given the continuous outcome measures, linear multilevel regression models were used, nesting individuals (level 1) within their intersectional social strata (level 2).

Two models were specified for each of the three outcomes (mental health score 2014, mental health score 2021, and change in mental health score 2014–2021). The comparison of the two models for each outcome allows for drawing conclusions about potential intersectional interaction effects. First, a null model, specifying no fixed effects (Model 1), was fit to estimate the total variance in the mental health outcome across two levels (i.e., between and within intersectional social strata). This model can be written as:

Model 1, null model: $\:{y}_{ij}={\beta\:}_{0}\:+\:{u}_{j\:}+\:{e}_{ij}$

In this formula, $\:{y}_{ij}$ denotes the mental health score (or change in mental health score) of individual *i* (*i* = 1, …, n_j_) in intersectional social stratum *j* (*j* = 1, …, *J*). β_0_ denotes the intercept, $\:{u}_{j\:}$ denotes the stratum-level residual, and $\:{e}_{ij}$ denotes the individual-level residual. The overall intercept ($\:{\beta\:}_{0})$ estimates the (change in) mental health status for the full sample (across all strata), and it is interpretable as a precision weighted grand mean. The stratum-specific intercepts (given by $\:{\beta\:}_{0}\:+\:{u}_{j\:}$) provide the predicted mental health in each stratum *j*. The stratum-level residual ($\:{u}_{j\:}$) therefore captures how stratum *j*’s predicted mental health score differs from the overall mental health score in the full sample. The individual-level residual ($\:{e}_{ij\:}$) captures the difference between the mental health of each individual and the average for their social stratum. The stratum-level residuals are assumed to follow a normal distribution with a mean of 0 and a variance of $\:{\sigma\:}_{u}^{2}$ around the overall population change in mental health.

$$\:{u}_{j}\:\sim\:{N\:(0,\:\sigma\:}_{u}^{2})$$

It should be noted that $\:{u}_{j}$ is not model a parameter, instead, $\:{u}_{j}$ is a shrunken residual estimated for each stratum using posterior Bayesian estimation techniques. This shrinkage, particularly of estimates for smaller intersectional social strata, limits the influence of outliers and produces more reliable and stable estimates than conventional approaches, such as single-level regression models with interaction effects [34–37].

Similar to the stratum-level residual, the individual-level residual effects are also assumed to follow a normal distribution with a mean of 0 and a variance of $\:{\sigma\:}_{e}^{2}$.

$$\:{e}_{ij}\:\sim\:{N\:(0,\:s}_{e}^{2})$$

The Variance Partition Coefficient (VPC) was calculated as follows:

$$\eqalign{& VPC = variance\,between\,social\,strata/ \cr & (variance\,between\,social\,strata + \cr & + variance\,within\,social\,strata) \times 100\% \cr & = {{\sigma _u^2} \over {\sigma _u^2 + \sigma _e^2}} \times 100\% \cr}$$

The VPC represents the percent of total variation ($\:{s}_{u}^{2}\:+\:{s}_{e}^{2}$) in the dependent variable that can be attributed to the between-stratum level. The VPC is a measure of discriminatory accuracy (DA), and in the null model it also provides a summary measure of inequality between strata standardized by the amount of ‘background’ variation in the outcome (including at the individual level). A low VPC indicates that outcomes are similar between intersectional strata but different within each intersectional group, which may imply that population-based approaches addressing the social determinants of mental health inequalities are justified. A high VPC could indicate a need for targeted interventions towards specific intersectional social strata at risk of poor mental health, alongside population-focused approaches, in line with the principles of proportionate universalism [38].

A second model (Model 2) was fit that included all additive main effects as dummy variables.

Model 2, main effects model:

$$\eqalign{& {y_{ij}} = {\beta _0} + {\beta _1}LowEducatio{n_j} + {\beta _2}IntermediateEducatio{n_j} \cr & + {\beta _3}LowIncom{e_j} + {\beta _4}IntermediateIncom{e_j} + {\beta _5}YoungAg{e_j} \cr & + {\beta _6}MiddleAg{e_j} + {\beta _7}Femal{e_j} + \>{u_{j\>}} + \>{e_{ij}} \cr}$$

In Model 2, the stratum level residual represents the “total intersectional interaction effect” for each stratum, namely the difference between the final predicted score (including any intersectional interaction effects) and the predicted score based on additive effects alone. If the stratum-level residual is (close to) 0, it indicates that the sum of the additive effects explain the between-stratum variance well for this outcome. If the stratum-level residual is significantly different from 0, it implies that the additive effects do not fully explain the existing inequalities, and something unique or extreme may be happening in that stratum to produce an outcome that breaks the overall, typical additive pattern. Thus Model 2 is thus fit for two reasons: (1) to generate final estimates for all strata for each outcome (final estimates include fixed and residual random effects), and (2) to evaluate the extent to which inequalities are patterned according to consistent additive patterns.

The Proportional Change in Variance (PCV) provides a measure that addresses this latter use of Model 2. Specifically, the PCV is the percent of the total between-stratum variance from the null model (Model 1) that could be explained after adjusting for additive main effects (as done in Model 2), using:

$$\:\text{P}\text{C}\text{V}\:=\:\frac{{\sigma\:}_{u,\:Model1}^{2}-\:{\sigma\:}_{u,\:Model2}^{2}}{{\sigma\:}_{u,\:Model1}^{2}}\:\times\:\:100\text{\%}$$

The higher the PCV score, the more of the total between-stratum variance of the null model can be explained by the additive main effects, and thus the more consistently additive the patterning of between-stratum inequalities is.

The MAIHDA models were run in MLwiN 3.06 [39], called from Stata 17.0 through the *runmlwin* command [40]. Bayesian Markov Chan Monte Carlo (MCMC) techniques were used to assess significance with 95% credible intervals with diffuse priors. The analysis was based on previously developed code [41]. The MCMC initialization values were determined using quasi-likelihood methods. Visual diagnostics of model convergence were performed for each outcome in Stata at a length of 10,000 iterations, a burn-in of 2,500 iterations and thinning every iteration. These showed that the models converged well, and for the final models, a burn-in of 5000 iterations and total length of 50,000 iterations (with thinning every 50 iterations) was used. Figures were created in Rstudio using the ggplot2 package [42]. The code fitting the MAIHDA models is provided in the online Supplement.

## Results

### Sample characteristics

The characteristics of the sample are presented in Table 1. Nearly half of the participants were highly educated (48.8%) and the largest group of participants had a high level of income (41.8%). Participants were somewhat more likely to be female (52.5%), and most participants (45.4%) were between 45 and 65 years old. The mean mental health score decreased by 1.0 point between 2014 (mean mental health score of 74.4) and 2021 (mean mental health score of 73.4).

**Table 1:** Descriptive statistics of the study sample and paired t-test results indicating significance of mental health changes, based on complete data *n* = 1157

Intersectional social strata dimensions	*N* (%)	Mental health score 2014	Mental health score 2021	Mean mental health change^1^ and confidence interval	t-value	Degrees of freedom	*p*-value
**Total sample**	1157 (100%)	74.4	73.4	-1.0* (-1.8 – -0.2)	2.4	1156	0.02
**Educational attainment (2014)**							
Low	297 (25.7%)	73.7	71.8	-1.9* (-3.7 – -0.1)	2.1	296	0.04
Middle	295 (25.5%)	73.4	72.8	-0.6 (-2.2–1.0)	0.7	294	0.47
High	565 (48.8%)	75.3	74.6	-0.7 (-1.8–0.3)	1.4	564	0.18
**Income level (2014)**							
Low	356 (30.8%)	70.2	69.4	-0.8 (-2.4–0.8)	1.0	355	0.33
Middle	449 (38.8%)	75.2	74.4	-0.8 (-2.1–0.5)	1.2	448	0.24
High	352 (30.4%)	77.7	76.2	-1.5* (-2.8 – -0.2)	2.2	351	0.03
**Gender (2014)**							
Female	608 (52.5%)	72.5	72.1	-0.4 (-1.6–0.7)	0.8	607	0.46
Male	549 (47.5%)	76.4	74.8	-1.6* (-2.8 – -0.5)	2.8	548	0.01
**Age (2014)**							
25–39 years old	243 (21.0%)	72.9	72.2	-0.7 (-2.4–1.1)	0.8	242	0.44
40–64 years old	525 (45.4%)	73.7	74.3	0.6 (-0.6–1.8)	-1.0	524	0.31
65 and older	389 (33.6%)	76.2	72.9	-3.3* (-4.8 – -1.9)	4.6	388	0.00

*denotes statistical significance of paired samples t-tests based on the 95% confidence interval

### Descriptive social gradients in mental health scores

Table 1 shows that in 2014, the mental health score of those with a low educational attainment was on average 1.6 points lower than the mental health score of those with a high educational attainment. These inequalities in mental health between education levels grew slightly, to a difference of 2.8 points by 2021, since specifically those with a low educational attainment experienced substantial mental health declines. Those with high income levels had a mean mental health score in 2014 of 7.5 points higher than the mental health of those with a low income level, this inequality somewhat decreased to a 6.8 point difference in 2021, mainly due to larger mental health setbacks among those with high incomes compared to those with low and intermediate income levels.

On average, women had a 3.9 points lower mental health score than men in 2014, this inequality slightly decreased to 2.7 points in 2021, due to stronger mental health declines among men than women. Those who were 65 or older had 3.3 points higher mental health score in 2014 compared to those under 40. In 2021, the mental health of those over 65 declined more rapidly than the mental health of the younger age groups, to a score of those over 65 that was merely 0.7 points higher than the mental health of those under 40, implying the previous mental health advantage of older age nearly disappeared.

Overall, the largest setbacks in mental health between 2014 and 2021 were experienced by those who were 65 and older in the 2014 survey (-3.3 points), those who had a low educational attainment (-1.9 points), men (-1.6 points), and among those with a high income level (-1.5 points).

### Distribution of participants across intersectional social strata

Across the 53 intersectional social strata, sample sizes ranged from one participant to 66 participants (see Table 2). Simulation studies have shown that MAIHDA is capable of producing reliable estimates at smaller sample sizes than conventional methods, potentially allowing disaggregation into the *N* = 5–10 range [37, 43, 44]. 47 intersectional social strata (88.7%) in this sample had N of five or larger and 40 strata (75.5%) had N of 10 or larger.

**Table 2:** Sample sizes of intersectional social strata defined as a combination of educational attainment, income level, age, and gender, *n* = 53

Sample size	Number of intersectional social strata	% of intersectional social strata
1–4	6	11.3%
5–9	7	13.2%
10–14	9	17.0%
15–19	7	13.2%
20–24	5	9.4%
25–29	4	7.6%
=>30	15	28.3%
Total	53	100%

### Results of MAIHDA models

In the null model for 2014 mental health (Table 3, Model 1 A), the VPC showed that 5.0% of the total variance could be attributed to between-stratum differences. Including the additive main effects (Table 3, model 1B) revealed general patterns of lower mental health scores among individuals with lower incomes, younger ages, and women. Although a small education gradient in mental health was identified, this effect was not statistically significant overall in additive terms. Overall, the VPC of Model 2 was reduced to 0.1%, indicating that most of the between-stratum inequalities observed in Model 1 A were accounted for by additive inequality patterns. Thus, relatively little remained to be explained by interaction effects. This was confirmed by the PCV of 97.8%. When examined individually, none of the 53 intersectional social strata experienced significant intersectional interaction effects in their mental health, meaning that the inequalities in mental health are well characterized by additive patterns.

In 2021, between-stratum intersectional inequalities in mental health were somewhat reduced compared to the earlier 2014 data, with a VPC of 3.1% (Table 3, Model 2A). Upon including the additive main effects (Table 3, Model 2B), the VPC reduced to 0.1% and the PCV was 95.4%, again indicating that the majority of the between-stratum inequalities are patterned additively. Inequalities based on main effects were identified among women and individuals with a low income. Educational attainment remained non-significantly associated with mental health, and age was no longer significant. None of the intersectional social strata experienced significant intersectional interaction effects in mental health, based on their educational attainment, income, age, and gender.

Examination of intersectional inequalities in the change in mental health between 2014 and 2021 revealed a VPC of 0.3%, indicating that, to the extent that strata experienced changes in mental health status, there were fairly modest between-stratum *differences* in those changes. Importantly, the non-zero VPC value implies some reshuffling of rank in the inequality distribution between strata, as well as a tendency toward a change of mental health scores. Upon adding the additive main effects (Model 3B), the VPC reduced to 0.2%, indicating that approximately half (PCV = 55.3%) of the between-strata variance could be explained by the main additive effects. While none of the intersectional social strata examined experienced statistically significant intersectional interaction effects in their change in mental health score, this is likely due to the small degree of change between waves overall. The PCV of 55.3% indicates some evidence of reshuffling that is not purely additive.

To better understand how inequality patterns changed between waves, we can examine the additive main effects from Model 3B. Educational attainment remained non-significantly associated with changes in mental health, and the income inequalities identified in 2014 and 2021 did not significantly change over time. Although women experienced disadvantages in mental health compared to men in 2014 and 2021, men experienced a larger, yet non-significant, decline between these years. A notable pattern stood out regarding how different age groups experienced changes in mental health between waves. Overall, individuals aged over 65 in 2014 experienced mental health declines from 2014 to 2021. Pre-pandemic (in 2014), those over 65 experienced an advantage in terms of their mental health, this advantage reduced over time to a mere modest advantage during the pandemic in 2021. Compared with the age group over 65 years old, the strata with those under 40 years old also experienced mental health declines between 2014 and 2021, though not as severely. The middle strata aged between 40 and 65 years old remained fairly stable in mental health scores between waves, though there was evidence of a small decrease as well. See Appendix 2 for an overview of the mental health trajectories over time for each social stratum.

**Table 3:** MAIHDA mental health estimates (and credibility intervals) with participants clustered by intersectional social strata

	Mental health score 2014		Mental health score 2021		Change in mental health score (2014 to 2021)	
	Null model (1 A)	Main Effects Model (1B)	Null model (2 A)	Main Effects Model (2B)	Null model (3 A)	Main Effects Model (3B)
	*N* = 1,157	*N* = 1,157	*N* = 1,157	*N* = 1,157	*N* = 1,157	*N* = 1,157
**Fixed effects**	Estimate* (95% CI)	Estimate* (95% CI)	Estimate* (95% CI)	Estimate* (95% CI)	Estimate* (95% CI)	Estimate* (95% CI)
Intercept	73.9* (72.5–75.2)	81.2* (79.0–83.5)	73.0* (71. 7–74.2)	77.1* (74.7–79.5)	-1.0* (-1.9 – -0.2)	-4.1* (-6.3 – -1.8)
Low educational attainment		-0.8 (-3.3–1.6)		-1.1 (-3.7–1.5)		-0.3 (-2.7–2.0)
Intermediate educational attainment		-0.7 (-2.9–1.2)		-0.6 (-3.0–1.4)		0.1 (-2.1–1.9)
High educational attainment		Reference		Reference		Reference
Low income level		-6.9* (-9.0 – -4.6)		-6.3* (-8.6– -3.9)		0.6 (-1.5–2.8)
Intermediate income level		-2.3* (-4.4 – -0.2)		-1.8 (-4.0–0.5)		0.5 (-1.5–2.5)
High income level		Reference		Reference		Reference
Young age (25–39)		-3.8* (-6.3 – -1.3)		-1.3 (-4.0–1.4)		2.5* (0.0–4.9)
Middle age (40–64)		-2.6* (-4.7 – -0.5)		1.2 (-1.1–3.4)		3.8* (1.7–5.8)
Old age (65+)		Reference		Reference		Reference
Female		-2.9* (-4.6 – -1.1)		-1.9* (-3.7 – -0.1)		1.0 (-0.6–2.8)
Male		Reference		Reference		Reference
**Random effects**						
Between stratum variance (level 2)	11.1 (3.3–24.3)	0.2 (0.0–1.8)	7.2 (0.1–17.3)	0.4 (0.0–2.8)	0.6 (0.00–4.4)	0.3 (0.0–2.3)
Within stratum variance (level 1)	210.3 (193.1–228.2)	207.8 (192.1–225.2)	237.7 (218.2–257.6)	236.1 (218.2–255.8)	195.0 (179.5–210.6)	193.2 (178.5–209.5)
VPC in %	5.0%* (1.5–10.4)	0.1%* (0.0–0.8)	3.1%* (0.00–7.1)	0.1%* (0.0–1.2)	0.3%* (0.0–2.3)	0.2%* (0.0–1.2)
PCV in %		97.8%		95.4%		55.3%

*Notes*: 95% CI = 95% Credible Interval. VPC = Variance Partition Coefficient. PCV = Proportional Change in Variance

*denotes statistical significance based on the 95% CI

**Fig. 1:**
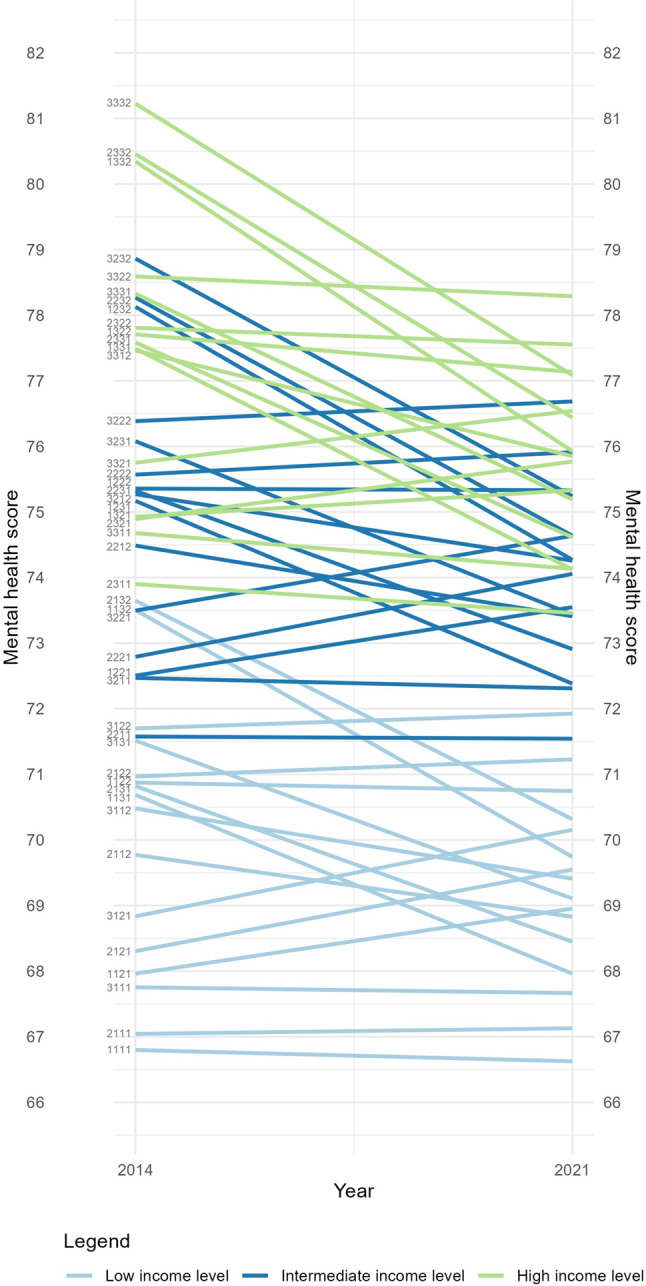
Mental health scores in 2014 and in 2021 per social stratum, based on MAIHDA estimates, stratified by income level. Social stratum ID’s are presented on the left side of the graphs, the first digit represents educational attainment (1: low, 2: intermediate, 3: high), the second digit represents income level (1: low, 2: intermediate, 3: high), the third digit represents age group (1: 25–40, 2: 40–65, 3: >65), and the final digit represent gender (1: female, 2: male). [to be printed in color]

**Fig. 2:**
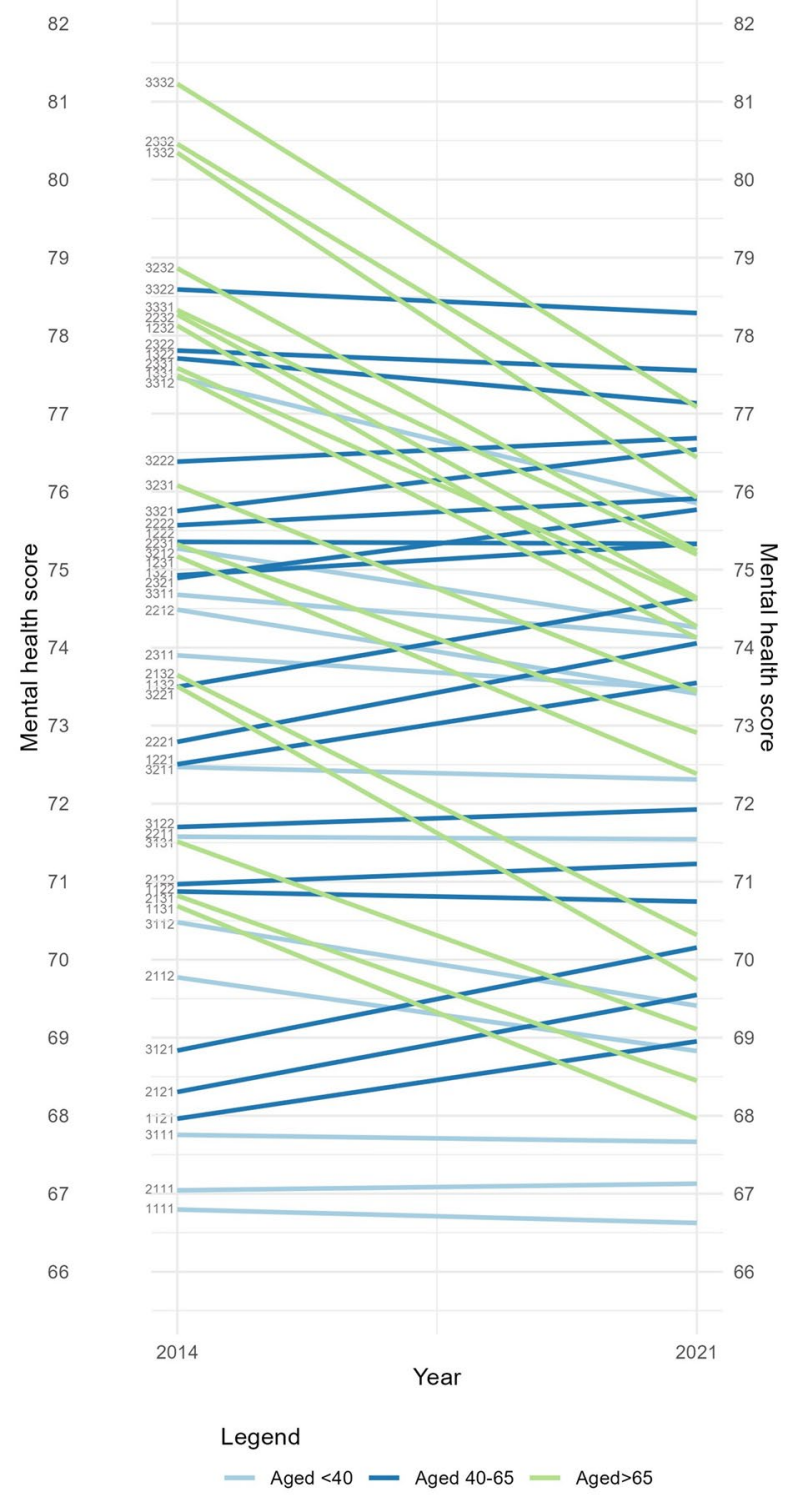
Mental health scores in 2014 and in 2021 per social stratum, based on MAIHDA estimates, stratified by age group. Social stratum ID’s are presented on the left side of the graphs, the first digit represents educational attainment (1: low, 2: intermediate, 3: high), the second digit represents income level (1: low, 2: intermediate, 3: high), the third digit represents age group (1: 25–39, 2: 40–64, 3: 65 and older), and the final digit represent gender (1: female, 2: male). [to be printed in color]

It can be difficult to conclude anything definitive about the intersectional patterning of inequalities based solely on summary statistics (VPC and PCV) or additive parameters. Therefore, to better understand the intersectional patterns, we provide visualizations of the mental health scores and trajectories for each stratum in Figs. 1 and 2. In Fig. 1, stratum lines are colored by income level and in Fig. 2 they are colored by age to highlight trajectory differences. Similar Figures distinguishing educational attainment and gender are available in Appendix 3. The Figures include the 47 intersectional social strata with sufficient sample sizes ( > = 5).

Figure 1 shows a clear income gradient in mental health. Participants in intersectional social strata with higher incomes fared better in terms of mental health compared to participants in strata with lower incomes. Among the social strata with lower-income participants, some experienced declines between waves, while participants in other lower-income strata experienced improvements. Although participants in intermediate and high-income strata had higher mental health scores overall, they were more likely to experience declines than those in lower-income strata. In 2021, there was a narrowing of between-stratum inequalities (with VPC dropping from 5.0 to 3.1%) and a reshuffling of rankings. However, the general pattern persisted: participants in higher-income strata still maintained higher mental health scores, despite experiencing more frequent mental health declines.

Figure 2 shows that intersectional social strata with older participants fared relatively well in 2014, yet experienced relatively large declines in mental health in 2021, bringing them closer to population means. It also shows that those aged 40 to 65 fared somewhat better in mental health than both younger and older strata, trending toward smaller declines in mental health between waves. Importantly, Figs. 1 and 2 demonstrate that though we can summarize a general trend for strata, such as those in the middle age group (aged over 40 and under 65) declining only slightly, there are actually important differences for individual strata. For example, among strata in the middle age group, some experienced meaningful improvements in mental health scores and others experienced substantial declines in mental health between waves. Though this averages out to a small decline overall, the intersectional patterns are distinct and worthy of examination.

## Discussion

This study explored intersectional inequalities in mental health in the Netherlands in 2014 (pre-COVID-19), 2021 (during COVID-19), and in mental health changes between 2014 and 2021, based on participants’ unique combinations of educational attainment, income level, gender, and age. The observed inequalities in mental health were largely patterned additively by income, gender, and age, but no educational inequalities were found. In 2014, lower mental health scores were experienced by people with lower and intermediate incomes compared to those with high income levels, by people of younger and middle age compared to those who are older, and by women compared to men. In 2021, lower mental health scores tended to be experienced by those with the lowest income level compared to those with intermediate and high income levels, and women compared to men.

Although other trends over time may have affected participants between 2014 and 2021, a main difference between these years was that the 2021 data captured potential mental health impacts of the COVID-19 pandemic and associated measures. During data collection in 2021, a wide range of restrictions were being (re-)introduced in the Netherlands, such as closures of all non-essential stores, social distancing, and cancellation of social events. As such, the 2021 mental health data likely captures how the pandemic itself, as well as strict control measures, may have impacted mental health. When exploring inequalities in mental health changes in the overall sample between 2014 and 2021, a small overall decrease of one point in mental health score (on a scale of zero to one hundred) was found. This small sample average decrease can obscure effects in different directions, because some intersectional social strata experienced more substantial declines over time, whereas others experienced average improvements in mental health. Between survey waves, there was a general “compression” of between-stratum inequalities, as seen in the VPC dropping from 5.0% in 2014 to 3.1% in 2021, and observable in Figs. 1 and 2. Visual inspection showed that this was explained by a decline in mental health scores among those who had the best mental health in 2014, and a relatively stable mental health among those who were initially at the bottom. One of the most notable trends between waves was the decline in mental health over time among those strata aged 65 and older in 2014. Younger age groups, including those < 40 years old and those aged 40–65 in 2014, tended to experience declines in mental health from 2014 to 2021, but the decline was less steep among the middle age strata.

The inequalities in mental health in 2014, 2021, and in the changes between the two waves were mainly patterned additively by income, gender, and age. This does not mean that the mental health inequalities are not intersectional in nature, but rather that the inequalities that exist, and which we understand to be produced through intersectional social processes, are patterned in more “predictable” (additive) ways, rather than with strata standing out from general patterns in specific and unique ways. The magnitude of these inequalities, and the precise nature of their changes over time, are still best understood through an intersectional framework and visualizations such as those provided in Figs. 1 and 2. For instance, if we had examined only the overall change in the sample between 2014 and 2021, we might have concluded that the COVID-19 pandemic (and other changes that occurred simultaneously between data waves) had only a modest negative impact on mental health. However, examined intersectionally, we see that there were notable trends and changes in inequalities across intersectional social strata.

A previous study that used interaction terms (rather than a multilevel approach) to predict intersectional mental health inequalities during the pandemic showed that women with lower levels of income in the Netherlands were more likely to encounter depressive symptoms [45], which is in line with the groups identified at risk of lower mental health scores in this study. A prior study explored intersectional inequalities in mental health pre-COVID-19 using MAIHDA among adolescents [46], examining similar intersections to those included in this study. Similar to our study, they found that pre-COVID-19 inequalities in mental health among adolescents across a range of countries, including the Netherlands, were also mainly explained by the additive effects of socioeconomic position, gender, and age.

In line with the absent effects of educational attainment on mental health as identified by Reep and Huskens, and Snel et al. [17, 19] and the conflicting educational effects identified by Gibson et al. [16], we identified nonsignificant educational gradients in mental health in 2014 and 2021, and a slight increase in educational inequalities over time. Similar to Snel et al. [19], we found income level to be a more important predictor of mental health in 2014 and 2021 than educational attainment. Those with lower incomes disproportionally faced many risks for mental health that persisted pre- and during COVID-19, such as poor and insecure housing and a lack of financial resources [19, 20]. These risks may have been more pronounced for those with lower incomes compared to those with lower educational attainments. Although educational inequalities slightly widened over time, income inequalities somewhat narrowed. This was likely explained by those with high incomes experiencing larger mental health setbacks over time compared to those with lower incomes. It has been suggested that those with lower incomes, possibly due to the structural disadvantages they face [47, 48], exhibit higher levels of optimism and resilience in coping with adverse circumstances [49].

Although women remained more disadvantaged in their mental health than men in 2014 and 2021, we found no evidence for the widening gender inequalities in mental health during the COVID-19 pandemic as suggested by previous studies [21, 22]. Instead, although women experienced lower mental health scores than men across both waves in this sample, men experienced a slightly stronger mental health decline over time than women. This difference might arise from gender differences in dealing with unemployment and economic setbacks resulting from COVID-19, which has been suggested to have a stronger effect on the emotional state of men than women [50].

In line with Dutch National Statistics [17], we found that those below the age of 40 experienced disadvantages in their mental health compared to the older age groups. Those over the age of 65 showed mental health advantages compared to other age groups in both waves, which was in line with National Statistics [17] and a study conducted in China [51]. However, in this longitudinal sample, those over the age of 65 experienced the largest mental health setbacks over time. This could possibly be explained by the elevated fear of suffering long-lasting health issues or even dying from a COVID-19 infection, as well as the loss of loved ones (such as spouses and partners) who were of a similar age. During the pandemic, those over the age of 60 were five times more likely to die from a COVID-19 infection compared to those under 60 [51]. In addition, older population groups likely faced more physical barriers to online socialization activities than younger age groups [52].

Our results highlight the relevance of a proportionate universalism approach for mental health, as suggested by Fisk et al. [53]. In the delivery of public interventions, proportionate access of populations with higher prevalence of mental health problems needs to be ensured. Depending on which cut-off point is used to consider an intersectional social stratums’ mental health score into likely mental health problems, different intersectional social strata may be especially at risk of poor mental health. Statistics Netherlands uses a score of 60 or lower to indicate mental health issues [54]. Although on average, all intersectional social strata are above this threshold, especially those strata in the 60 and early 70 range likely contain many individuals below this threshold. A score below 72 has also been found to reliably indicate mental health issues among the Dutch population [30]. This cut-off would imply that all intersectional social strata with lower incomes could on average be expected to experience increased mental health issues. This highlights the necessity of proportionately addressing the persisting income inequalities in mental health, and underlines the necessity of accessible mental health support for especially those with low income levels. Those with lower incomes in the Netherlands experience many barriers, such as financial barriers, in relation to mental health support [55]. Moreover, increased socioeconomic security and a better outlook towards increased quality of life, by for instance ensuring sufficient income and suitable housing, are vital for mental health [56].

In interpreting the results, several strengths and limitations of this study need to be taken into account. This study utilized longitudinal panel data, which enabled us to follow mental health of the same participants over time. This allowed us to study inequalities in changes over time in addition to cross-sectional inequalities, which offered a broader picture of inequalities in mental health. Furthermore, MAIHDA holds several advantages over conventional regression and interaction-based methods for intersectional analyses, such as an easier interpretation of model results. The MAIHDA approach also adjusts estimates based on sample size, providing more accurate results than alternative approaches when samples are smaller [34, 35, 57]. Close to 90% of social strata included in our data had sample sizes of five participants or more, and over 75% of the social strata had sample sizes of ten participants or more. Simulation studies have shown that MAIHDA can reliably produces estimates for strata even with very small samples (e.g., 5 participants per stratum) [37, 43, 44], making this method particularly useful to explore intersectional inequalities in relatively small samples, such as in this study. Although MAIHDA likely produced reliable estimates in our small sample, the relatively small sample sizes of social strata may have limited our ability to identify strata that “stood out” in terms of having intersectional interaction effects.

A potential limitation is that it is likely that the sample reflected some healthy participant bias, since healthy participants may be more likely to participate in surveys [58]. However, as the overall mental health trend in this sample was in line with national statistics for the Netherlands [17], as well as findings of comparative international research [59], this may not have greatly impacted our results. Further, given the seven-year timeframe from 2014 to 2021, it is likely that participants had other experiences in addition to COVID-19 that may have shaped their mental health, including aging, changing health status, or impact from historical events (political, economic, and similar). Moreover, the intersectional social strata were based on 2014 reported data on income, education, gender, and age. We did not control for changes in income or other factors that may have shaped mental health during the COVID-19 pandemic, such as co-habitation status, job loss, or caregiving responsibilities. Appendix 4 offers some descriptives regarding income changes over time, but since these descriptives are inconclusive, the effect of income changes on mental health over time should be examined in future research, as well as the effects of other relevant factors such as job loss and family status.

Another limitation is that we were unable to explore additional axes of inequality due to constraints imposed by the size and composition of the dataset. For instance, we were unable to estimate outcomes for non-binary gendered respondents (as the 2014 survey only measured gender in binary terms). Additionally, the lack of ethnic diversity within the sample prevented us from exploring outcomes for different ethnicities. Specific age groups that have been shown to have a high risk of developing poor mental health during COVID, such as those between the ages of 18 and 25 [17], or institutionalized elderly [60], were also not represented in the sample. More specific age groups have been included in a study conducted in the UK context [61]. This study showed that the youngest age groups (generations Y and Z), and the oldest age group (the silent generation) especially experienced mental health setbacks, while baby boomers experienced mental health improvements [61]. The mental health of younger age groups warrants more attention. Future research could also examine potential intersectional income inequalities after 2022 and their impact on mental health. The financial COVID-19 support issued by the Dutch government may have prevented income inequalities from widening during the pandemic. If this is the case, a delayed mental health setback could be anticipated in relation to the recovery of COVID-19 related loans, which started in 2022.

## Conclusions

This study explored intersectional inequalities in mental health in 2014, 2021, and in changes between 2014 and 2021 based on differences in educational attainment, income level, gender, and age. Using an intersectional analysis approach, such as MAIHDA, allows for the consideration of diverse and heterogeneous experiences along multiple axes of social inequalities simultaneously, even when sample sizes are small [44, 62]. Furthermore, MAIHDA can reveal important variations in inequality patterns that, using traditional approaches, would remain largely unseen. Mental health inequalities were mainly patterned additively by income, gender, and age. More effort needs to be made to improve the mental health of disadvantaged population groups, notably people with low incomes, women, and young adults. Given the high risk of poor mental health among social strata consisting of people with low incomes, enhanced income protection alongside equitable access to mental health care may improve their well-being. In future public health crisis responses, measures need to be taken to protect the mental health of those over the age of 65 years.

## Electronic supplementary material

Below is the link to the electronic supplementary material.

Supplementary Material 1

Supplementary Material 2: Appendices 1 – 4

## Data Availability

The dataset generated and/or analyzed during the current study is not publicly available due to privacy regulations, but can be accessed on a secure server on reasonable request. Such requests can be made via globe.study@erasmusmc.nl.

## Abbreviations

MAIHDA: Multilevel Analysis of Individual Heterogeneity and Discriminatory Accuracy
GLOBE: Dutch acronym for Health and Living Conditions of the Population of Eindhoven and surroundings
ISCED: International Standard Classification of Education
