# Supplementary material for: Intersectional inequalities in mental health by education, income, gender, and age before and during the COVID-19 pandemic in the Netherlands: a longitudinal study
Source: Int J Equity Health. 2024 Nov 25;23:250. doi: 10.1186/s12939-024-02338-6 (PMC11590372; doi:10.1186/s12939-024-02338-6)
Supplement: Supplementary file 2 — Supplementary Material 2: Appendices 1 – 4 [file 12939_2024_2338_MOESM2_ESM.doc]

**Appendix 1: Sample selection**

Figure 1 presents a flowchart of the sample selection.

*Figure 1:* Flowchart of sample selection

Initial Dataset

GLOBE participants who responded to 2014 & 2021 surveys

(n = 1,354)

Eligible (n = 1,354)

Excluded due to failure to meet age check / unlikely to be filled in by the same respondent based on age:

- Missing age in 2014 or 2021: -10
- Difference in age between 2014 and 2021 >2 standard deviations from the mean: -12

Excluded due to missing data on:

- Educational attainment 2014: -5
- Household equivalent income level 2014: -138
- Mental health in 2014 or 2021: -32

Analytic sample for imputed sensitivity analyses (n = 1,332)

Analytic sample for MAIHDA analyses (n = 1,157)

First, we conducted a check to ensure the survey was filled in by the same person in 2014 and 2021, by looking if a person’s age logically increased between 2014 and 2021. This resulted in dropping 10 participants with missing ages in 2021, and dropping 12 participants whose change in age was more than two standard deviations away from the mean age change. Next, 5 participants with a missing educational attainment, and 138 participants with a missing income level were excluded. Since only those participants were included who filled in at least three out of five items of the MHI-5 scale to determine mental health score, 32 participants were deleted for missing data on mental health. This resulted in a final size of N=1157 (implying that 14.5% of participants of the original sample of N=1354 were excluded).

Imputation was used to test if the results were sensitive to missing data. Multiple imputation using chained equations was used. All variables used in the analyses were used to inform imputation, and additional auxiliary variables were living with a partner (yes or no) and employment status (in paid employment or not). Ten imputed datasets were created, in line with the percentage of missing data on the variable with the most missing data (income level). Due to processing limitations, we were unable to run the main MAIHDA models on imputed datasets and thus deviated from the pre-registration. Instead, we followed (Holman et al., 2022)’s example and compared the point estimates of main regression models using imputed and non-imputed datasets. Since the large majority of inequalities were due to main effects, this approach can reliably capture potential differences in main effects between imputed and unimputed datasets. The results (Table 1) indicated no significant differences between the point estimates. Given the comparable findings, we expect the MAIHDA estimates to be unaffected by the missing data.

*Table 1*. Main effect regression model comparisons based on multiply imputed and unimputed datasets

| **Fixed effects** | Unimputed | Imputed | Unimputed | Imputed | Unimputed | Imputed |
| --- | --- | --- | --- | --- | --- | --- |
| Estimate (Credible interval)* | Mental health 2014 | | Mental health 2021 | | Change in mental health | |
|  | N = 1,157 | N = 1,332 | N = 1,157 | N = 1,332 | N = 1,157 | N = 1,332 |
| Intercept | 67.0 (64.1 - 69.8)* | 66.8 (64.1 - 69.5)* | 66.7 (63.7 - 69.7)* | 66.0 (63.0 - 68.9)* | -0.3 (-3.0 - 2.5) | -0.8 (-3.4 - 1.8) |
| Low education | Reference | Reference | Reference | Reference | Reference | Reference |
| Intermediate education | 0.1 (-2.3 - 2.5) | 0.4 (-1.8 - 2.7) | 0.4 (-2.1 - 3.0) | 0.9 (-1.5 - 3.3) | 0.3 (-2.0 - 2.6) | 0.5 (-1.7 - 2.6) |
| High education | 0.8 (-1.5 - 3.1) | 0.7 (-1.4 - 2.9) | 1.0 (-1.4 - 3.5) | 0.9 (-1.4 - 3.2) | 0.2 (-2.0 - 2.5) | 0.2 (-1.9 - 2.3) |
| Low income level | Reference | Reference | Reference | Reference | Reference | Reference |
| Intermediate income level | 4.6 (2.5 - 6.6)* | 4.9 (2.7 - 7.0)* | 4.5 (2.3 - 6.7)* | 5.0 (2.7 - 7.2)* | -0.0 (-2.0 - 1.9) | -0.2 (-2.1 - 1.7) |
| High income level | 6.8 (4.6 - 9.1)* | 7.2 (5.0 - 9.4)* | 6.3 (3.9 - 8.7)* | 6.7 (4.2 - 9.2)* | -0.6 (2.7 - 1.6) | -0.6 (-2.7 - 1.5) |
| Young age (25-39) | Reference | Reference | Reference | Reference | Reference | Reference |
| Middle age (40-64) | 1.1 (-1.1 - 3.4) | 0.9 (-1.3 - 3.0) | 2.4 (0.1 - 4.8)* | 2.2 (-0.1 - 4.6) | 1.3 (-0.9 - 3.5) | 1.4 (-0.6 - 3.5) |
| Old age (65+) | 3.7 (1.2 - 6.2)* | 3.3 (0.9 - 5.7)* | 1.2 (-1.4 - 3.5) | 0.7 (-1.9 - 3.3) | -2.5 (-4.9 - -0.1)* | -2.5 (-4.8 - -0.1)* |
| Female | Reference | Reference | Reference | Reference | Reference | Reference |
| Male | 2.9 (1.2 - 4.6)* | 3.2 (1.6 - 4.8)* | 1.9 (0.1 - 3.7)* | 3.0 (1.2 - 4.7)* | -1.0 (-2.6 - 0.6) | -0.3 (-1.8 - 1.3) |

**Appendix 2: Overview of MAIHDA mental health trajectory estimates for all social strata**

| **Stratum** | **Label** Educational level, Income level, Age group, Gender | **Sample size** | **Mental health score 2014  and credible interval** | **Mental health score 2021  and credible interval** | **Change in mental health score and credible interval** |
| --- | --- | --- | --- | --- | --- |
| 1111 | Low edu, Low inc, <40, F | 5 | 66.8 (63.5 - 70.2) | 66.6 (63.3 - 70.2) | -0.2 (-3.1 - 3.2) |
| 1112 | Low edu, Low inc, <40, M | 4 | 69.6 (66.3 - 73) | 68.2 (64.6 - 71.9) | -1.5 (-4.8 - 1.8) |
| 1121 | Low edu, Low inc, 40-65, F | 28 | 68 (65.4 - 70.5) | 69 (66.2 - 71.7) | 0.9 (-1.6 - 3.5) |
| 1122 | Low edu, Low inc, 40-65, M | 19 | 70.9 (68 - 73.8) | 70.7 (67.6 - 73.7) | -0.3 (-3.3 - 2.3) |
| 1131 | Low edu, Low inc, >65, F | 52 | 70.7 (68.3 - 73.1) | 68 (65.4 - 70.6) | -2.8 (-5.1 - -0.4) |
| 1132 | Low edu, Low inc, >65, M | 32 | 73.5 (70.9 - 76) | 69.7 (66.9 - 72.4) | -3.9 (-6.4 - -1.4) |
| 1212 | Low edu, Mid inc, <40, M | 2 | 74.3 (70.8 - 77.9) | 72.9 (69.2 - 76.8) | -1.5 (-4.9 - 2) |
| 1221 | Low edu, Mid inc, 40-65, F | 17 | 72.5 (69.6 - 75.1) | 73.5 (70.4 - 76.4) | 1 (-1.7 - 3.7) |
| 1222 | Low edu, Mid inc, 40-65, M | 16 | 75.4 (72.6 - 78) | 75.3 (72.4 - 78.2) | -0.1 (-2.8 - 2.6) |
| 1231 | Low edu, Mid inc, >65, F | 40 | 75.2 (72.6 - 77.6) | 72.4 (69.6 - 75) | -2.8 (-5.2 - -0.4) |
| 1232 | Low edu, Mid inc, >65, M | 26 | 78.1 (75.6 - 80.6) | 74.3 (71.6 - 76.9) | -4 (-6.5 - -1.6) |
| 1311 | Low edu, High inc, <40, F | 1 | 73.7 (70.2 - 77.3) | 72.8 (68.9 - 76.6) | -1 (-4.6 - 2.5) |
| 1312 | Low edu, High inc, <40, M | 1 | 76.5 (73.1 - 80.1) | 74.6 (70.8 - 78.4) | -2 (-5.4 - 1.5) |
| 1321 | Low edu, High inc, 40-65, F | 14 | 74.9 (72.1 - 77.8) | 75.3 (72.2 - 78.4) | 0.3 (-2.5 - 3.1) |
| 1322 | Low edu, High inc, 40-65, M | 9 | 77.7 (74.6 - 80.8) | 77.1 (73.8 - 80.4) | -0.7 (-3.7 - 2.3) |
| 1331 | Low edu, High inc, >65, F | 19 | 77.5 (74.8 - 80.3) | 74.1 (71.2 - 77.2) | -3.4 (-6.1 - -0.6) |
| 1332 | Low edu, High inc, >65, M | 12 | 80.3 (77.6 - 83) | 75.9 (73 - 78.8) | -4.5 (-7.2 - -1.9) |
| 2111 | Mid edu, Low inc, <40, F | 16 | 67 (64.1 - 69.7) | 67.1 (63.9 - 70) | 0.1 (-2.8 - 2.7) |
| 2112 | Mid edu, Low inc, <40, M | 8 | 69.8 (66.5 - 72.9) | 68.8 (65.3 - 72.1) | -1 (-3.9 - 2.1) |
| 2121 | Mid edu, Low inc, 40-65, F | 38 | 68.3 (65.9 - 70.6) | 69.5 (67 - 72) | 1.2 (-1.1 - 3.5) |
| 2122 | Mid edu, Low inc, 40-65, M | 23 | 71 (68.3 - 73.5) | 71.2 (68.3 - 73.9) | 0.2 (-2.4 - 2.6) |
| 2131 | Mid edu, Low inc, >65, F | 16 | 70.8 (68.2 - 73.5) | 68.4 (65.6 - 71.5) | -2.3 (-4.9 - 0.4) |
| 2132 | Mid edu, Low inc, >65, M | 12 | 73.6 (70.8 - 76.3) | 70.3 (67.3 - 73.4) | -3.4 (-6.1 - -0.6) |
| 2211 | Mid edu, Mid inc, <40, F | 8 | 71.6 (68.6 - 74.4) | 71.5 (68.4 - 74.5) | 0 (-2.9 - 2.7) |
| 2212 | Mid edu, Mid inc, <40, M | 7 | 74.5 (71.4 - 77.7) | 73.4 (70.1 - 76.9) | -1.1 (-4.2 - 2) |
| 2221 | Mid edu, Mid inc, 40-65, F | 39 | 72.8 (70.5 - 75) | 74.1 (71.5 - 76.4) | 1.2 (-1.2 - 3.4) |
| 2222 | Mid edu, Mid inc, 40-65, M | 26 | 75.6 (73 - 78) | 75.9 (73.1 - 78.5) | 0.3 (-2.3 - 2.7) |
| 2231 | Mid edu, Mid inc, >65, F | 11 | 75.3 (72.6 - 77.9) | 72.9 (70 - 75.7) | -2.4 (-5 - 0.2) |
| 2232 | Mid edu, Mid inc, >65, M | 23 | 78.3 (75.5 - 80.9) | 74.6 (71.6 - 77.5) | -3.6 (-6.5 - -1) |
| 2311 | Mid edu, High inc, <40, F | 7 | 73.9 (70.8 - 76.7) | 73.5 (70.1 - 76.5) | -0.5 (-3.5 - 2.3) |
| 2312 | Mid edu, High inc, <40, M | 4 | 76.7 (73.5 - 79.9) | 75.2 (71.7 - 78.6) | -1.5 (-4.7 - 1.6) |
| 2321 | Mid edu, High inc, 40-65, F | 22 | 74.9 (72.1 - 77.4) | 75.8 (72.9 - 78.5) | 0.8 (-1.7 - 3.4) |
| 2322 | Mid edu, High inc, 40-65, M | 9 | 77.8 (75.1 - 80.5) | 77.6 (74.6 - 80.5) | -0.3 (-3 - 2.3) |
| 2331 | Mid edu, High inc, >65, F | 12 | 77.6 (74.8 - 80.5) | 74.6 (71.6 - 77.8) | -3 (-5.6 - 0) |
| 2332 | Mid edu, High inc, >65, M | 14 | 80.5 (77.8 - 83.3) | 76.4 (73.5 - 79.5) | -4.1 (-6.8 - -1.3) |
| 3111 | High edu, Low inc, <40, F | 22 | 67.8 (65.1 - 70.3) | 67.7 (64.7 - 70.4) | 0 (-2.7 - 2.4) |
| 3112 | High edu, Low inc, <40, M | 13 | 70.5 (67.5 - 73.3) | 69.4 (66.4 - 72.4) | -1 (-3.7 - 1.8) |
| 3121 | High edu, Low inc, 40-65, F | 34 | 68.8 (66.4 - 71.3) | 70.2 (67.6 - 72.8) | 1.3 (-1 - 3.8) |
| 3122 | High edu, Low inc, 40-65, M | 20 | 71.7 (69.1 - 74.3) | 71.9 (69.2 - 74.7) | 0.2 (-2.3 - 2.8) |
| 3131 | High edu, Low inc, >65, F | 10 | 71.5 (68.7 - 74.5) | 69.1 (66.1 - 72.4) | -2.3 (-5.1 - 0.7) |
| 3132 | High edu, Low inc, >65, M | 4 | 74.3 (71.6 - 77.4) | 70.8 (67.7 - 74.1) | -3.5 (-6.3 - -0.5) |
| 3211 | High edu, Mid inc, <40, F | 40 | 72.5 (70 - 74.8) | 72.3 (69.6 - 74.9) | -0.1 (-2.7 - 2.3) |
| 3212 | High edu, Mid inc, <40, M | 34 | 75.3 (72.9 - 77.7) | 74.3 (71.7 - 76.9) | -1 (-3.3 - 1.4) |
| 3221 | High edu, Mid inc, 40-65, F | 55 | 73.5 (71.3 - 75.6) | 74.6 (72.3 - 76.8) | 1.2 (-1 - 3.1) |
| 3222 | High edu, Mid inc, 40-65, M | 66 | 76.4 (74.5 - 78.3) | 76.7 (74.6 - 78.8) | 0.3 (-1.5 - 2.2) |
| 3231 | High edu, Mid inc, >65, F | 11 | 76.1 (73.5 - 78.7) | 73.4 (70.5 - 76.3) | -2.6 (-5.2 - 0.1) |
| 3232 | High edu, Mid inc, >65, M | 28 | 78.9 (76.5 - 81.3) | 75.2 (72.7 - 77.8) | -3.6 (-5.8 - -1.3) |
| 3311 | High edu, High inc, <40, F | 37 | 74.7 (72.3 - 77.1) | 74.1 (71.6 - 76.7) | -0.5 (-2.8 - 1.8) |
| 3312 | High edu, High inc, <40, M | 34 | 77.5 (74.9 - 79.9) | 75.9 (73.1 - 78.5) | -1.6 (-4.1 - 0.8) |
| 3321 | High edu, High inc, 40-65, F | 35 | 75.8 (73.3 - 78) | 76.5 (74 - 79.1) | 0.8 (-1.5 - 3.2) |
| 3322 | High edu, High inc, 40-65, M | 55 | 78.6 (76.5 - 80.9) | 78.3 (76 - 80.8) | -0.3 (-2.4 - 1.9) |
| 3331 | High edu, High inc, >65, F | 19 | 78.3 (75.7 - 80.8) | 75.2 (72.4 - 77.8) | -3.1 (-5.6 - -0.7) |
| 3332 | High edu, High inc, >65, M | 48 | 81.2 (79 - 83.5) | 77.1 (74.7 - 79.6) | -4.2 (-6.5 - -1.9) |

**Appendix 3: Figures for educational attainment and gender**

No mental health inequalities were identified based on educational attainment. Figure 1 shows much variation in mental health changes over time across social strata with different educational attainments.

Figure 2 visualized the social strata by gender. Figure 2 shows that the intersectional social strata of men fared relatively well in 2014, yet experienced relatively larger declines in their mental health in 2021, bringing them closer to the female means. Although the mental health of men declined at a faster pace, women still experienced an overall mental health disadvantage compared to men.


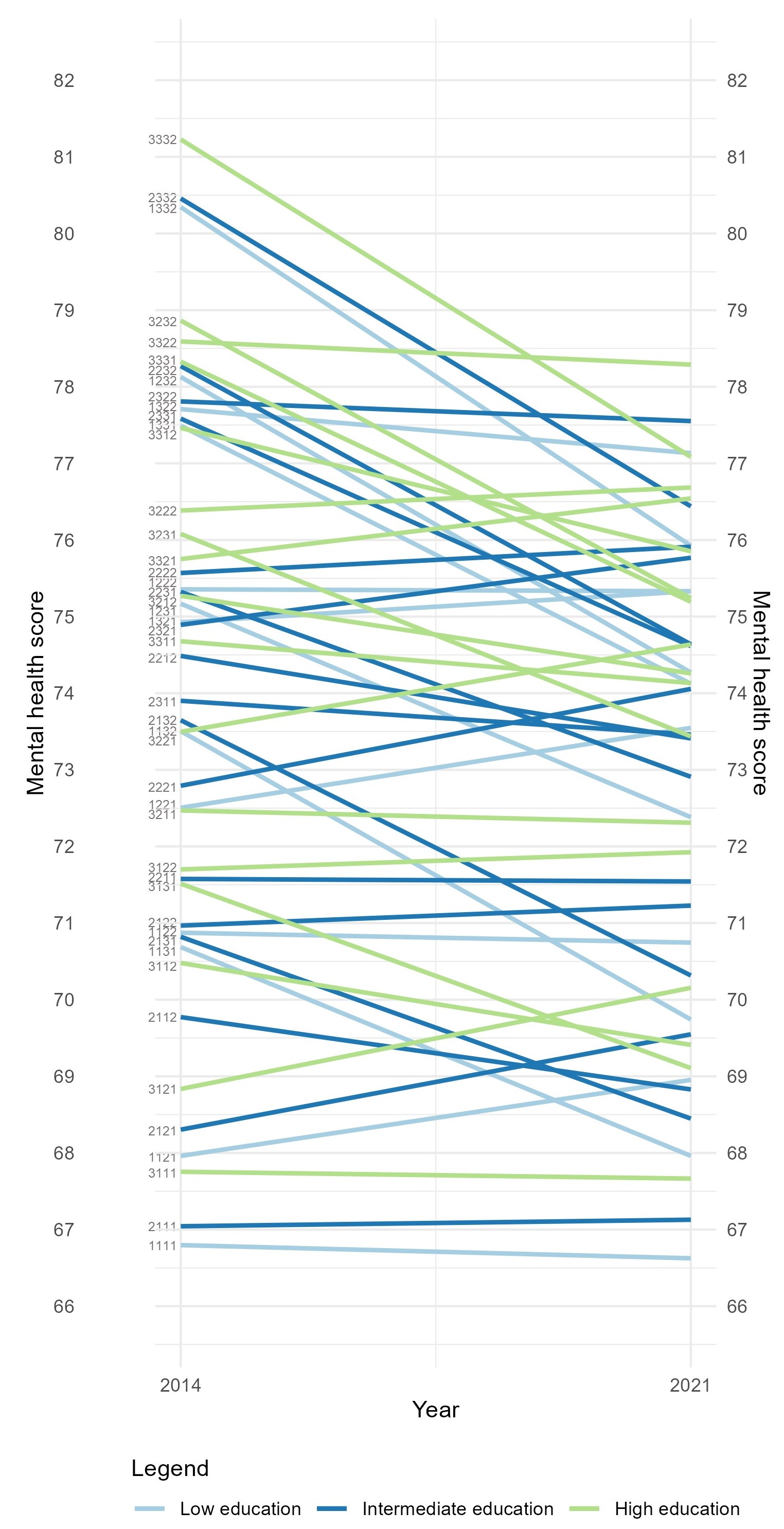


*Figure 1:* Mental health scores in 2014 and in 2021 per social stratum, based on MAIHDA estimates, stratified by educational attainment. Social stratum ID’s are presented on the left side of the graphs, the first digit represents educational attainment (1: low, 2: intermediate, 3: high), the second digit represents income level (1: low, 2: intermediate, 3: high), the third digit represents age group (1: 25-40, 2: 40-65, 3: >65), and the final digit represent gender (1: female, 2: male). [to be printed in color]


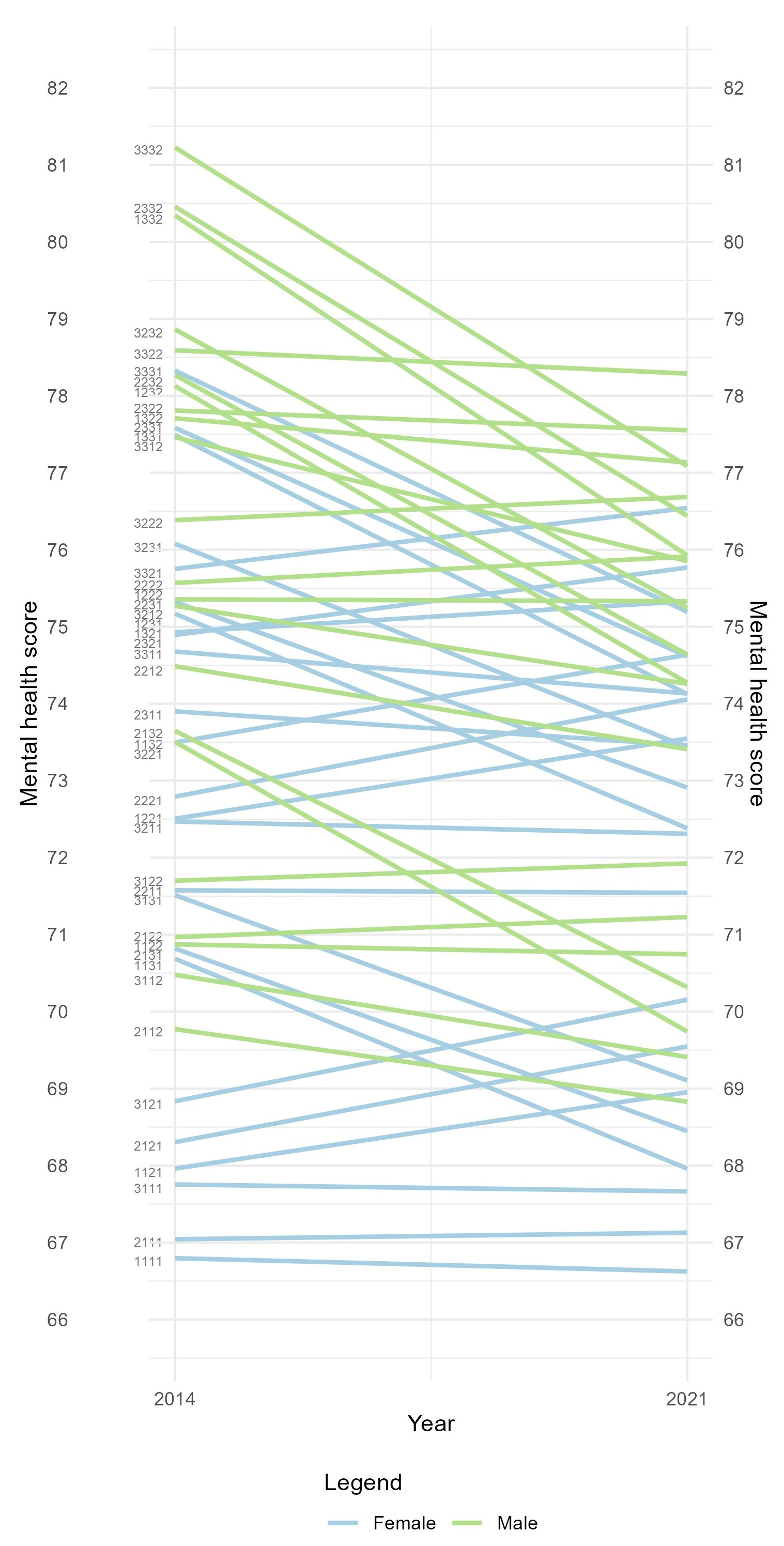


*Figure 2:* Mental health scores in 2014 and in 2021 per social stratum, based on MAIHDA estimates, stratified by gender. Social stratum ID’s are presented on the left side of the graphs, the first digit represents educational attainment (1: low, 2: intermediate, 3: high), the second digit represents income level (1: low, 2: intermediate, 3: high), the third digit represents age group (1: 25-40, 2: 40-65, 3: >65), and the final digit represent gender (1: female, 2: male). [to be printed in color]

**Appendix 4: Supplementary descriptives**

The intersectional social strata explored in this study are formed based on 2014 data for educational attainment, income level, gender, and age, to explore potential intersectional inequalities in mental health changes between 2014 and 2021. Although minor changes have been self-reported between 2014 and 2021, the variables used to form the intersectional social strata remained relatively stable between 2014 and 2021 (only few participants reported a change in their gender, age changed at the same speed for all participants –those who did not meet this criterium were deleted, and education-nal attainment was relatively stable since the sample includes participants aged 25 and older who often have completed their education). The exception to this is income level. Since income is likely to vary over time, it is possible that mental health changes could result from changes in income.

We therefore provided additional descriptive overviews of changes in income level per social stratum, and the observed scores for change in mental health. In addition, the GLOBE 2021 survey included three items capturing self-perceived changes due to COVID-19 (either due to COVID-19 measures, lockdowns, or the disease); whether participants experienced financial set-backs due to COVID-19, whether participants experienced physical health set-backs due to COVID-19, and whether participants experienced mental health set-backs due to COVID-19. Descriptive information for each of these variables is provided for the top 5 highest and lowest intersections.

Based on descriptive data, intersectional social strata with larger decreases in mental health seemed to experience somewhat smaller income increases based on the self-reported household income data, compared to those with the largest increases in mental health. However, it should be noted that the data presented in the Table combine items that relate to different timeframes. The MHI-5 items in 2014 and 2021 were based on one’s perception of mental health over the last month. The same applies to the income difference, which was based on the reported household income in 2014 and 2021. However, the three COVID-19 related items asked respondents to respond based on their perception of the change *due to COVID-19.* Since the survey was taken in November of 2021, participants likely responded based on the situation between March 2020 (the start of the COVID-19 pandemic in the Netherlands) and November 2021. It is for instance a possibility that respondents initially experienced gains in income between 2014 and 2019, but experienced decreases in 2020-2021. This may explain why the self-reported income changes due to COVID-19 do not seem to capture the changes in reported household income across the both waves well. Moreover, the percentages presented here were based on small sample sizes and should be interpreted with caution.

| | **Intersectional social strata with the largest decreases in mental health** | | | | | | | | | | | | | | | | --- | --- | --- | --- | --- | --- | --- | --- | --- | --- | --- | --- | --- | --- | --- | |  |  |  |  |  |  | **Self-perceived change due to COVID-19 in…** | | | | | | |  |  | | **Social** |  | **Mental health score** | | | **Income** | **Income** |  |  | **Mental health** | | | **Physical Health** | | | | **stratum** | **n** | **2014** | **2021** | **Change** | **Change** | **Improved** | **Stable** | **Decreased** | **Improved** | **Stable** | **Decreased** | **Improved** | **Stable** | **Decreased** | | 2232 | 23 | 79.65 | 71.65 | -8.00 | 321.12 | 0% | 87.3% | 21.7% | 0% | 87% | 13% | 0% | 65.2% | 34.8% | | 1332 | 12 | 80.33 | 72.42 | -7.92 | 147.75 | 16.7% | 83.3% | 0% | 0% | 91.7% | 8.3% | 0% | 75% | 25% | | 3231 | 11 | 72.18 | 64.73 | -7.46 | 85.56 | 0% | 100% | 0% | 10% | 70% | 20% | 0% | 70% | 30% | | 2212 | 7 | 77.71 | 70.71 | -7.00 | 550.00 | 14.3% | 85.7% | 0% | 0% | 71.4% | 28.5% | 0% | 85.7% | 14.3% | | 3331 | 19 | 76.21 | 70.84 | -5.37 | 13.56 | 0% | 100% | 0% | 5.3% | 73.7% | 21.1% | 0% | 78.9% | 21.1% |  | **Intersectional social strata with the largest increases in mental health** | | | | | | | | | | | | | | | | --- | --- | --- | --- | --- | --- | --- | --- | --- | --- | --- | --- | --- | --- | --- | |  |  |  |  |  |  | **Self-perceived change due to COVID-19 in…** | | | | | | |  |  | | **Social** |  | **Mental health score** | | | **Income** | **Income** |  |  | **Mental health** | | | **Physical Health** | | | | **stratum** | **n** | **2014** | **2021** | **Change** | **Change (2014-2021)** | **Improved** | **Stable** | **Decreased** | **Improved** | **Stable** | **Decreased** | **Improved** | **Stable** | **Decreased** | | 2321 | 22 | 71.27 | 75.45 | 4.18 | -89.66 | 18.2% | 81.8% | 0% | 4.5% | 72.7% | 22.7% | 13.6% | 72.7% | 13.6% | | 2311 | 7 | 78.29 | 82.48 | 4.19 | 13.51 | 60% | 20% | 20% | 20% | 60% | 20% | 0% | 100% | 0% | | 3131 | 10 | 69.40 | 74.00 | 4.60 | 154.23 | 0% | 99.9% | 11.1% | 11.1% | 77.8% | 11.1% | 0% | 100% | 0% | | 1221 | 17 | 68.24 | 73.88 | 5.65 | 173.05 | 12.5% | 75% | 12.5% | 0% | 87.5% | 12.5% | 0% | 87.5% | 12.5% | | 1111 | 5 | 52.80 | 70.40 | 17.60 | 626.20 | 20% | 80% | 0% | 0% | 100% | 0% | 0% | 60% | 40% | |
| --- | --- | --- | --- | --- | --- | --- | --- | --- | --- | --- | --- | --- | --- | --- | --- | --- | --- | --- | --- | --- | --- | --- | --- | --- | --- | --- | --- | --- | --- | --- | --- | --- | --- | --- | --- | --- | --- | --- | --- | --- | --- | --- | --- | --- | --- | --- | --- | --- | --- | --- | --- | --- | --- | --- | --- | --- | --- | --- | --- | --- | --- | --- | --- | --- | --- | --- | --- | --- | --- | --- | --- | --- | --- | --- | --- | --- | --- | --- | --- | --- | --- | --- | --- | --- | --- | --- | --- | --- | --- | --- | --- | --- | --- | --- | --- | --- | --- | --- | --- | --- | --- | --- | --- | --- | --- | --- | --- | --- | --- | --- | --- | --- | --- | --- | --- | --- | --- | --- | --- | --- | --- | --- | --- | --- | --- | --- | --- | --- | --- | --- | --- | --- | --- | --- | --- | --- | --- | --- | --- | --- | --- | --- | --- | --- | --- | --- | --- | --- | --- | --- | --- | --- | --- | --- | --- | --- | --- | --- | --- | --- | --- | --- | --- | --- | --- | --- | --- | --- | --- | --- | --- | --- | --- | --- | --- | --- | --- | --- | --- | --- | --- | --- | --- | --- | --- | --- | --- | --- | --- | --- | --- | --- | --- | --- | --- | --- | --- | --- | --- | --- | --- | --- | --- | --- | --- | --- | --- | --- | --- | --- | --- | --- | --- | --- | --- | --- | --- | --- | --- | --- | --- | --- | --- | --- | --- | --- | --- | --- | --- | --- | --- | --- | --- | --- | --- | --- | --- | --- | --- | --- | --- | --- | --- | --- | --- | --- | --- | --- | --- | --- | --- | --- | --- | --- | --- | --- | --- | --- | --- | --- | --- | --- | --- | --- | --- | --- | --- | --- | --- | --- |
